# Investigating the Role of Zebrafish Retinoschisin Homologs Rs1a and Rs1b During Retinal Development

**DOI:** 10.1002/dneu.23012

**Published:** 2025-10-31

**Authors:** Isa van der Veen, Céline Koster, Jacoline B. Ten Brink, Maarten Kamermans, Camiel J. F. Boon

**Affiliations:** ^1^ Department of Ophthalmology, Amsterdam UMC University of Amsterdam Amsterdam The Netherlands; ^2^ Department of Human Genetics Amsterdam UMC, Section Ophthalmogenetics University of Amsterdam Amsterdam The Netherlands; ^3^ The Netherlands Institute of Neuroscience (NIN) Royal Dutch Academy of Art & Science (KNAW) Amsterdam The Netherlands; ^4^ Department of Ophthalmology, Leiden University Medical Center Leiden University Leiden The Netherlands

**Keywords:** danio rerio, morpholino oligo, retinal development, retinoschisin, X‐linked juvenile retinoschisis

## Abstract

Deficiency in the retinoschisin protein (RS1) causes X‐linked juvenile retinoschisis (XLRS), a retinal degenerative disease that disrupts retinal layers and forms cystic cavities. In addition to its structural function, RS1 is believed to play a role in retinal development. A zebrafish model may provide insights into the role of Rs1 in the earliest stages of retinal development. To explore this, we created a zebrafish model with RS1 deficiency by knocking down the two homologs, Rs1a and Rs1b.

Gene expression and protein presence were assessed in Wildtype Tüpfel Longfin zebrafish at 1, 24, 48, 72, 96, and 120 h post‐fertilization (hpf). We then performed morpholino (MO)‐mediated knockdown targeting *rs1a* and *rs1b* mRNA, using scrambled oligos (SC) as controls. MOs or SCs were injected at the 1–4 cell stage, and samples were collected at 48, 72, 96, and 120 h post‐fertilization (hpf). The effects were analyzed using immunohistochemistry (IHC) and RNA sequencing.

Expression of *rs1a* and *rs1b* was first observed at 48 hpf. The successful knockdown of Rs1 was confirmed via IHC. At 72 hpf, Rs1 protein presence was eliminated without affecting overall embryo development. Transcriptional analysis showed enrichment of genes related to axon guidance at 72 hpf and visual perception at 96 hpf. On IHC, photoreceptor protein levels were lower in MO‐injected retinae at 96 and 120 hpf. Our findings align with those observed in rodent and organoid models for XLRS, demonstrate the potential of the zebrafish model for XLRS, and advocate for continued research on Rs1 in zebrafish.

## 1 Introduction

X‐linked juvenile retinoschisis (XLRS) is one of the most prevalent monogenic retinal degenerative diseases. Worldwide, 1:5000–1:25,000 males are affected, with symptoms starting as young as three months old (Molday et al. 2012; Hahn et al. 2022). Affected female homozygotes are rare, and heterozygous carriers are generally unaffected (Hahn et al. 2022). Patients present with cystoid macular cavities and splitting of the retinal layers in the macula and, in up to 50% of patients, in the peripheral retina (Hahn et al. 2022). Functionally, the b‐wave amplitude on electroretinogram is reduced (George et al. 1995). As a result of these retinal degenerative changes in the macula, patients are often unable to read, drive, or recognize faces. Some patients experience complications such as retinal detachment and vitreous hemorrhages due to peripheral retinal degenerative abnormalities (Hahn et al. 2022).

In 1997, the cause of XLRS was identified as a deficiency in the retinoschisin gene (*RS1*; OMIM#312700) (Sauer et al. 1997; The Retinoschisis Consortium 1998). While the precise role of the RS1 protein is unknown to date, it is likely to be involved in cellular adhesion and communication (Takada et al. 2008). The lack of functional RS1 protein in the retina leads to a breakdown in structural integrity and thereby causes the characteristic schisis phenotype of XLRS (Sauer et al. 1997). RS1 deficiency is also associated with impaired synaptic signaling between photoreceptors (PRs) and bipolar cells (BPC) (Ou et al. 2015). Hence, XLRS is often regarded as primarily a structural disease that results in functional aberrations through the breakdown of the normal retinal architecture. However, recent research suggests a primary role for RS1 in retinal functioning independent of morphological anomalies (Eleftheriou et al. 2022).

The precise function of RS1 is currently unknown, which can be attributed partly to the limitations of presently available and exclusively rodent models for XLRS (van der Veen et al. 2024). Although rodent retinae are available for ex vivo histological analyses, rodent eyes are difficult to access for in vivo imaging or intervention before eye‐opening. The processes that occur in this time period are of particular interest, as schisis cavities are already present in rats at eye opening (Zeng et al. 2022; Ye et al. 2022). For this purpose, zebrafish (*Danio rerio)* are an attractive alternative. Zebrafish are a rapid, easily accessible model organism for retinal development, because all developmental events occur in quick succession outside the maternal environment in a mostly transparent organism (Howe et al. 2013). The vertebrate retina is highly conserved; human, rodent, and zebrafish retinae share the same cellular layers containing the same cell types (Avanesov and Malicki 2010). Unlike rodents, zebrafish are diurnal and have a cone‐dominated retina. They have four different cone subtypes that allow them to discriminate red, green, blue, and (unlike human and rodent) ultraviolet (UV) wavelengths (Allison et al. 2004; Seritrakul and Gross 2019). Mutations in several retinal disease‐causing loci result in similar phenotypes in humans and zebrafish, including but not limited to pyruvate dehydrogenase deficiency and choroideremia (Taylor et al. 2004; Krock et al. 2007).

Despite its attractiveness as a model for retinal disease, a zebrafish model for XLRS has not been described before. Such a model could yield new insights in vivo into the processes that, in rodents, occur before eye‐opening. The *RS1* gene has two orthologs in zebrafish: *rs1a* and *rs1b*. Little is known about these homologs in terms of expression and protein localization, other than one report of *rs1* expression in two‐day‐old embryos (Yokoi et al. 2009). To be able to explore zebrafish as a model for XLRS, we first investigated the spatiotemporal expression profile of *rs1a* and *rs1b* in the early developing wildtype zebrafish retina. Subsequently, we created a temporary knockdown of *rs1a* and *rs1b* using morpholino oligos (MOs), a time‐ and cost‐effective approach to determine whether RS1 deficiency impacts the developing zebrafish retina in ways that align with established rodent findings.

## 2 Methods

### 2.1 Zebrafish Maintenance and Husbandry

Wild‐type zebrafish, *D. rerio*, Tüpfel Long Fin (TLF) strain, were maintained at 28°C under a 14/10 h light/dark cycle. Breeding fish were transferred to a breeding tank, and fertilized eggs were collected the following morning, 30 min–1 h after the room light was switched on. Embryos were grown in embryo medium (E3; 5 mM NaCl, 0.17 mM KCl, 0.33 mM CaCl_2_, 0.33 mM MgSO4, 0.1% methylene blue) at 28°C following ZFIN standard procedures, up to 120 h post‐fertilization (hpf).

### 2.2 MO Design and Microinjection

Two translation‐blocking MOs and two scrambled control oligos (SCs) were designed and synthesized with Gene Tools (LLC, Philomath, OR, USA) to simultaneously knockdown Rs1a and Rs1b protein translation. Design parameters, final oligo sequences, and the target region in the mRNA can be found in Supporting Information 1. The mRNA sequence at the oligo binding region was confirmed using Sanger sequencing BigDye Terminator v1.1 (Thermo Scientific, Waltham, MA, USA). The concentration of each oligo was determined such that it could consistently knockdown protein expression for up to 72 h on immunohistochemistry (IHC) without resulting in larval deformations (Figure S1C). At the chosen dose, deformed embryos were rare (<1% per hundred injections) and were excluded from further analysis, if present. Micro‐injection capillaries (Harvard Apparatus) were prepared by pulling the capillary in half and producing two tapered glass needles using a Flaming/Brown Pipette Puller Model P‐1000 (Sutter). Subsequently, the needles were trimmed under a microscope such that the required injection volume was produced with each pulse. The diluted MO was backloaded into the microinjection capillary using Eppendorf microloader pipette tips (Thermo Scientific, Waltham, MA, USA). Approximately 100 zebrafish embryos were placed into agarose embryo‐holding trays, which were made by placing a mold in 1.5% agarose in E3 to create grooves for the embyos to self‐align. The embryos were injected in the center of the yolk between the 1 and 4‐cell stage (approximately 1 hpf) with 500 pL of a 1:1 mixture containing either both MOs or both SCs and phenol red using a Pneumatic PicoPump pv280 (World Precision Instruments) and a Nikon SMZ800 microscope. After injection, embryos were maintained at 28°C in E3 for up to five days. At 24, 48, 72, 96, and 120 hpf, the fish were terminated by immersion in ice water and processed for further analysis. Larvae were inspected under a dissection microscope to check for MO overdose‐related deformations and, in rare cases (less than 1% of injected embryos), excluded from further analysis. Injections were performed in triplicate for each time point.

### 2.3 Cryosectioning and IHC

Embryos that had not yet left the chorion were manually dechorionated after termination. Whole embryos and larvae were washed in phosphate‐buffered saline (PBS) and fixed in 4% paraformaldehyde (PFA) in 1x PBS overnight at 4°C. Samples were cryopreserved in 25% and subsequently in 35% sucrose until sunken and were embedded in Tissue‐Tek Optimal Cutting Temperature (O.C.T.) Compound (Sakura, Torrance, CA, USA). Following, they were snap‐frozen in liquid N_2_ and stored at −80°C until sectioning. Whole fish were sectioned using a Cryostar cryostat in the midsaggital plane. Sections of 4 µm thickness were collected onto 10% poly‐l‐lysine‐coated SuperFrost microscope slides (Thermo Scientific, Waltham, MA, USA) in series of five and stored at −20°C until staining. For IHC, sections were thawed and rehydrated at room temperature (RT) for 1 h, then encircled with a liquid‐blocking protein pen (Daido Sangyo). Primary antibodies were incubated for 1.5 h at RT or 12 h overnight at 4°C in blocking buffer (1% bovine albumin serum [BSA] and 0.2% Triton X‐100 in 1x PBS). Primary antibody details and concentrations are listed in Table S1. The secondary antibody (goat‐anti‐rabbit Cy3 [1:200, 111‐166‐003, Jackson ImmunoResearch, Ely, UK] or donkey‐anti‐mouse Cy3 [1:200, 115‐166‐003, Jackson ImmunoResearch, Ely, UK]) was incubated with DAPI (1:400) in 0.2% Triton X‐100 in 1x PBS for 1 h at RT in the dark. Sections were embedded with ProLong Gold Antifade mounting medium (Thermo Scientific, Waltham, MA, USA). Stained sections were imaged on a Leica SP8 confocal microscope.

### 2.4 Quantification of Rs1

For quantification of Rs1 protein distribution across retinal layers, integrated fluorescence intensity was measured per layer (ONL, OPL, INL, IPL, GCL) using defined ROIs on immunohistochemically‐stained cryosections in ImageJ. The signal per layer was normalized to the total Rs1 signal within the retina of the same embryo, and expressed as percentage contribution per layer. Quantification was performed from 72 hpf onwards, as retinal lamination at 48 hpf was insufficiently developed to allow reliable segmentation. Quantitative data are presented as mean ± standard error of the mean (SEM).

### 2.5 RNA Isolation and RT‐PCR

Five whole zebrafish larvae were pooled and homogenized using a pestle in TRIzol reagent (Ambion). RNA was extracted and processed according to the manufacturer's protocol. The concentration and quality of the extracted RNA were evaluated using the Nanodrop following the manufacturer's protocol (ND‐1000). Complementary DNA (cDNA) was reverse transcribed using Superscript III (Invitrogen, Waltham, MA, USA) and an oligodT, following the supplier's protocol. RT‐PCR was performed with HOT FIREpol DNA Polymerase and specific primers to confirm mRNA presence (Solis Biodyne, Tartu, Estonia). The primers used are listed in Table S2.

### 2.6 Transcriptomic Analysis

Total RNA was extracted from MO‐ and SC‐injected larvae, as well as uninjected siblings, and triplicates were selected for RNA sequencing analysis. Each group contained at least 15 larvae. RNA yield, purity and integrity, cDNA library construction, and Illumina sequencing were performed by NovoGene (Cambridge, United Kingdom). Differentially expressed genes (DEGs) were identified as log_2_ fold change ←0.5;>0.5 and adjusted *p‐*value <0.05. Enrichment was performed using gene ontology (GO) and Kyoto Encyclopedia of Genes and Genomes (KEGG) pathway analyses. Pathways and terms were regarded as significantly enriched if the corrected *p*‐value reached <0.05.

### 2.7 Statistical Analysis

All experiments were conducted with at least three independent biological replicates from separate clutches per condition. Quantified Rs1 layer distributions were statistically compared across developmental time points using two‐way ANOVA followed by Tukey's post‐hoc tests. Normality and heterogeneity of variance were assessed prior to analysis. A *p*‐value of < 0.05 was considered statistically significant. All analyses were performed using GraphPad Prism version 8.4.0.

## 3 Results

### 3.1 The Human RS1 Protein Is Conserved in Zebrafish

**FIGURE 1:**
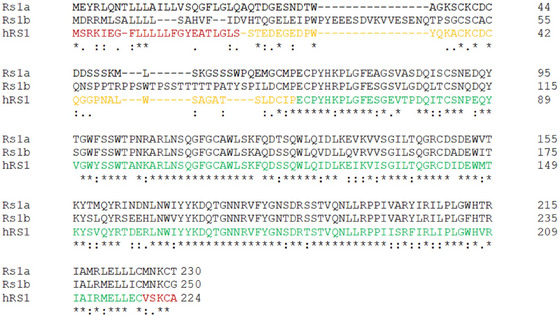
The alignments of the protein sequences of Rs1a (NP_001003438.1) and Rs1b (NP_001004655.1) with human orthologue RS1 (hRS1 (NP_000321.1)) show a high degree of homology between the three proteins. The alignment was performed using the multiple sequence alignment program Clustal Omega (version 1.2.2.) using default parameters. Rs1a and Rs1b have a similarity score to each other of 72.5% and to hRS1, respectively, 81.7% and 71.5%. Amino acids with identical physiochemical properties are taken into account for the determination of similarity. (*) denotes identical residues, conversed residues are indicated with (:), and similar residues with (.). RS1 = retinoschisin. In the sequence of hRS1, the protein's domains are indicated. Red = N‐terminal signal peptide and C‐terminal segment, orange = RS domain, green = discoidin domain.

To assess potential homology between human RS1 (hRS1), and zebrafish Rs1a and Rs1b, a Clustal Omega (version 1.2.2., 2022‐06‐01) multiple sequence alignment was performed. Similarity scores were obtained through the EMBOSS Needle pairwise sequence alignment program and yielded similarity scores of 72.5% (Rs1a to Rs1b), 81.7% (Rs1a to hRS1), and 71.5% (Rs1b to hRS1). The RS1 protein consists of an N‐terminal signal peptide and a C‐terminal segment flanking the RS domain, unique to and named after this protein, and the evolutionarily conserved discoidin domain, involved in extracellular communication (Vogel 1999) (Figure 1). Notably, a large degree of similarity between the proteins is found in the discoidin domain, which is thought to be crucial for the protein's function (The Retinoschisis Consortium 1998; Takada et al. 2008).

### 3.2 Expression of *rs1a* and *rs1b* in Zebrafish Embryos

**FIGURE 2:**
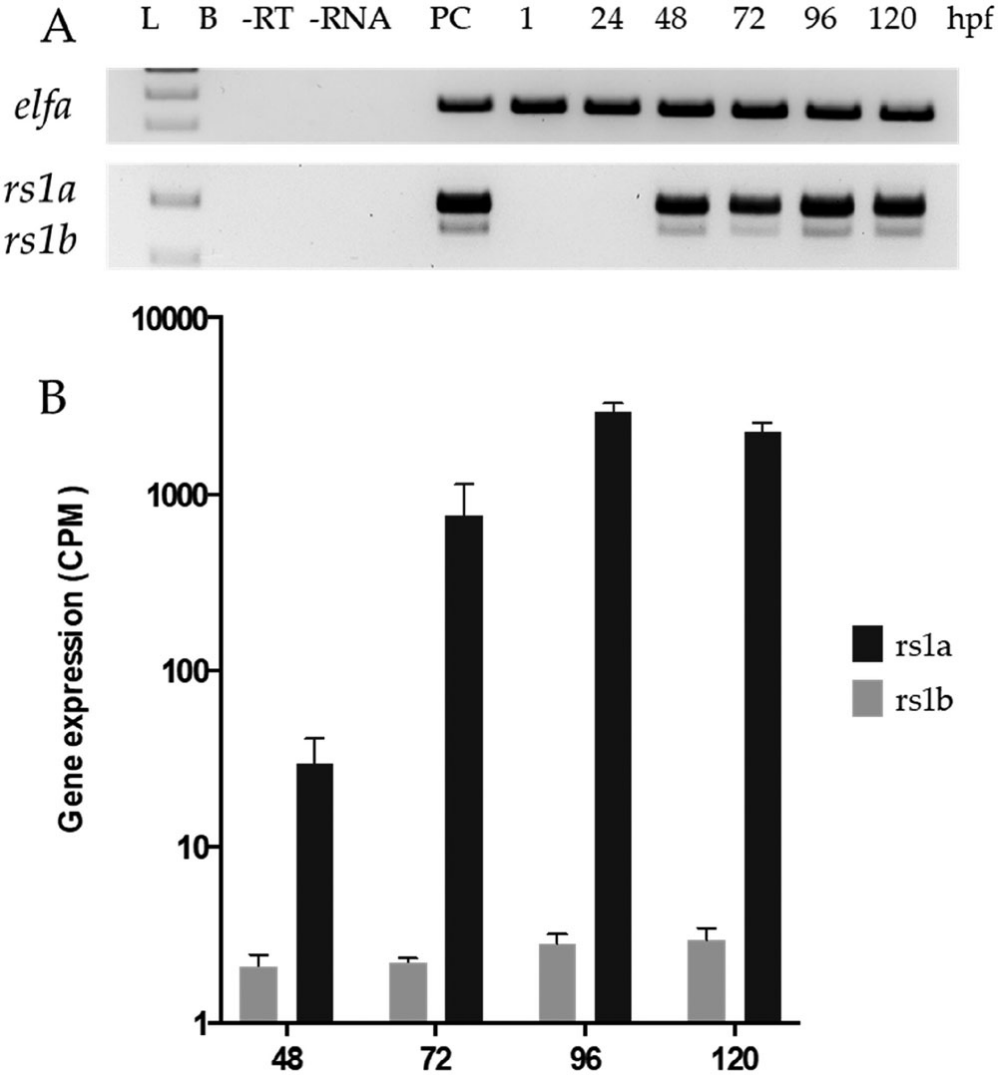
***rs1a* and *rs1b* are expressed in the developing zebrafish eye from 48 hpf onwards**. Gene expression was assessed at 1, 24, 48, 72, 96, and 120 hpf. A: The expression of reference gene elongating factor alpha (*elfa)* was consistent across all samples. Gene expressions of *rs1a* and *rs1b* were first detectable at 48 hpf and were detected throughout 120 hpf. B. Expression levels, as measured by RNA sequencing, of *rs1a* and *rs1b*. Hpf = hours post‐fertilization, B = blank,—RT = cDNA synthesis negative control without reverse transcriptase, ‐RNA = cDNA synthesis negative control without input RNA, PC = positive control with adult zebrafish eye cDNA, 1 = 1 hpf, 24 = 24 hpf, 72 = 72 hpf, 96 = 96 hpf, 120 = 120 hpf.

To assess when gene expression of *rs1a* and *rs1b* is first detectable, we performed RT‐PCR on embryos every 24 h between 1 and 120 hpf. Expression of *rs1a* and *rs1b* at 1, 24, 48, 72, and 96 hpf are shown in Figure 2A. For both genes, expression was first detected at 48 hpf and was maintained up to the last time point, sampled at 120 hpf. Counts per million per time point were derived from RNA sequencing (Figure 2B). *Rs1b* mRNA was found in considerably lower levels than *rs1a*.

### 3.3 Rs1a and Rs1b Protein Presence in the Developing Zebrafish Retina

**FIGURE 3:**
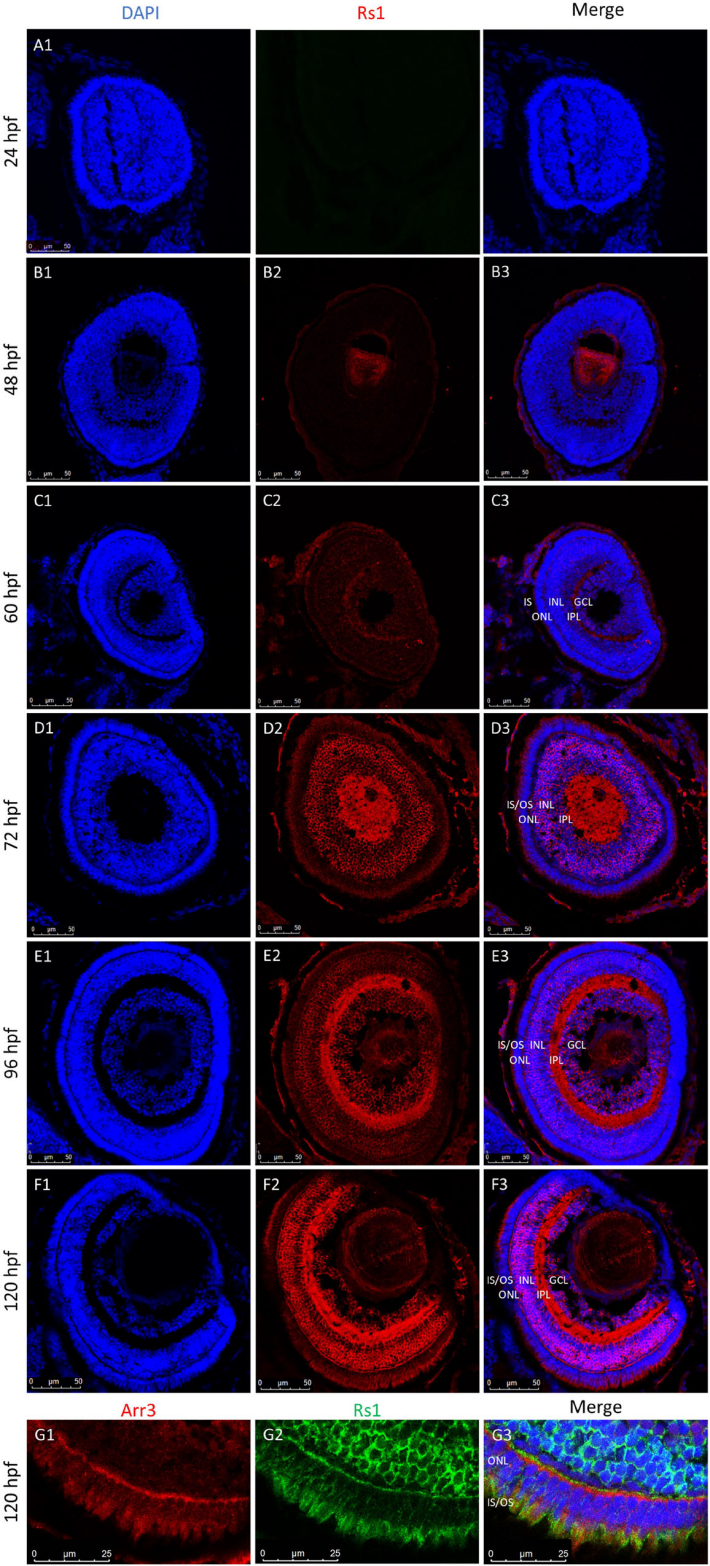
**The spatiotemporal overview of retinoschisin (Rs1) protein presence during the first five days of retinal development shows that Rs1 protein presence is first detected at 48 hpf and increases in intensity and spatial distribution up to 120 hpf**. Cryosections were incubated with primary antibodies overnight at 4°C. At least 3 biological replicates were examined per timepoint. A: No positive immunostaining of Rs1 was observed within the first 24 h of retinal development. B: At 48 h, Rs1 protein presence was first observed in this experiment in low levels in the ganglion cell layer (GCL). C: At 60 hpf, a weak signal was detected in the GCL, the inner plexiform layer (IPL), and the outer nuclear layer (ONL) D‐E. The intensity of Rs1 immunostaining continued to increase throughout 72 (D), 96 (E), and 120 (F) hpf. G. Co‐staining of Rs1 and cone arrestin (Arr3) demonstrated that Rs1 surrounds the developing outer segments at 120 hpf. F. Rs1 presence was quantified per retinal layer and normalized to total Rs1 signal per sample. Data represent mean percentage of Rs1 signal per layer (± standard error of the mean). A shift in Rs1 localization is observed from predominately INL at 72 hpf toward increasing IPL and GCL contribution at later stages. Hpf = hours post fertilization, Rs1 = retinoschisin, Arr3 = cone arrestin, DAPI = 4′,6‐diamidino‐2‐phenylindole.

A polyclonal RS1 antibody was used to detect Rs1a and Rs1b (together: Rs1) protein presence on IHC throughout retinal development. Rs1 protein presence could first be observed at low levels in the ganglion cell layer (GCL) at 48 hpf (Figure 3B), followed by the inner plexiform layer (IPL) and inner nuclear layer (INL) at 60 hpf (Figure 3C). Between 72 and 96 hpf, positive immunostaining had extended to the newly forming inner (IS) and outer segments (IS) layer, and increased in overall intensity (Figure 3D,E). At 120 hpf, staining was most pronounced in the IPL, surrounding the inner retinal cells in the INL, and among the PRs (Figure 3F). To pinpoint the precise localization of Rs1 protein relative to the PR cell bodies, we performed a co‐staining of Rs1 and cone arrestin (Arr3). At 96 and 120 hpf, Rs1 protein could be observed in the OPL and the IS, as well as surrounding the PR nuclei in the ONL and the developing OS (Figure 3G). Quantification of Rs1 signal showed the redistribution across retinal layers from 72 to 120 hpf. At all timepoints, the majority of signal was localized in the INL. The contribution from the IPL increased significantly over time (Figure 3F).

### 3.4 Validation of Rs1a and Rs1b Translation‐Blocking MO

**FIGURE 4:**
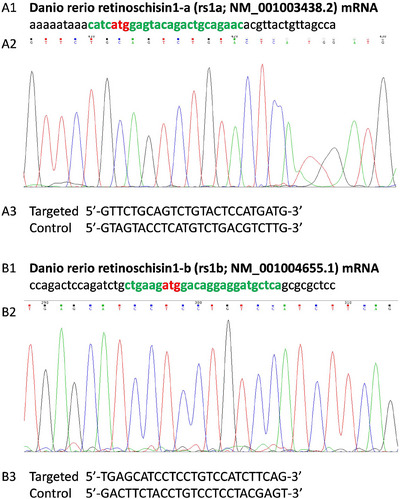
**The morpholino oligos (MO) for retinoschisin (*RS1*) homologs *rs1a* and *rs1b* were designed to block the translation of the mRNAs into proteins, and were developed alongside a control oligo (SC)**. A‐B: The mRNA sequence of the MO binding region was confirmed on Sanger sequencing for *rs1a* (A1) and *rs1b* (B1) to ensure perfect binding. A1‐B1: the first 50 nucleotides of the *Danio Rerio rs1a* (A1) and *rs1b* (B1) mRNA. The MO binding region is indicated in bold green text. Red indicates the start codon. A3‐B3: ‘'5’ to ‘'3’ sequence of the MO targeting *rs1a* (A3) and *rs1b* (B3) and their untargeted control oligos containing the same base pair ratios.

In order to determine the effect of Rs1 deficiency on retinal development, two MOs were designed to block the translation of *rs1a* and *rs1b*. To ensure perfect MO target binding, the target location was confirmed using Sanger sequencing (Figure 4). For the *rs1a* sequencing product, the last 6–10 base pairs were computationally determined with less certainty due to difficulties with primer selection caused by the poly‐A upstream of the start codon. Nevertheless, the bases can be distinguished.

**FIGURE 5:**
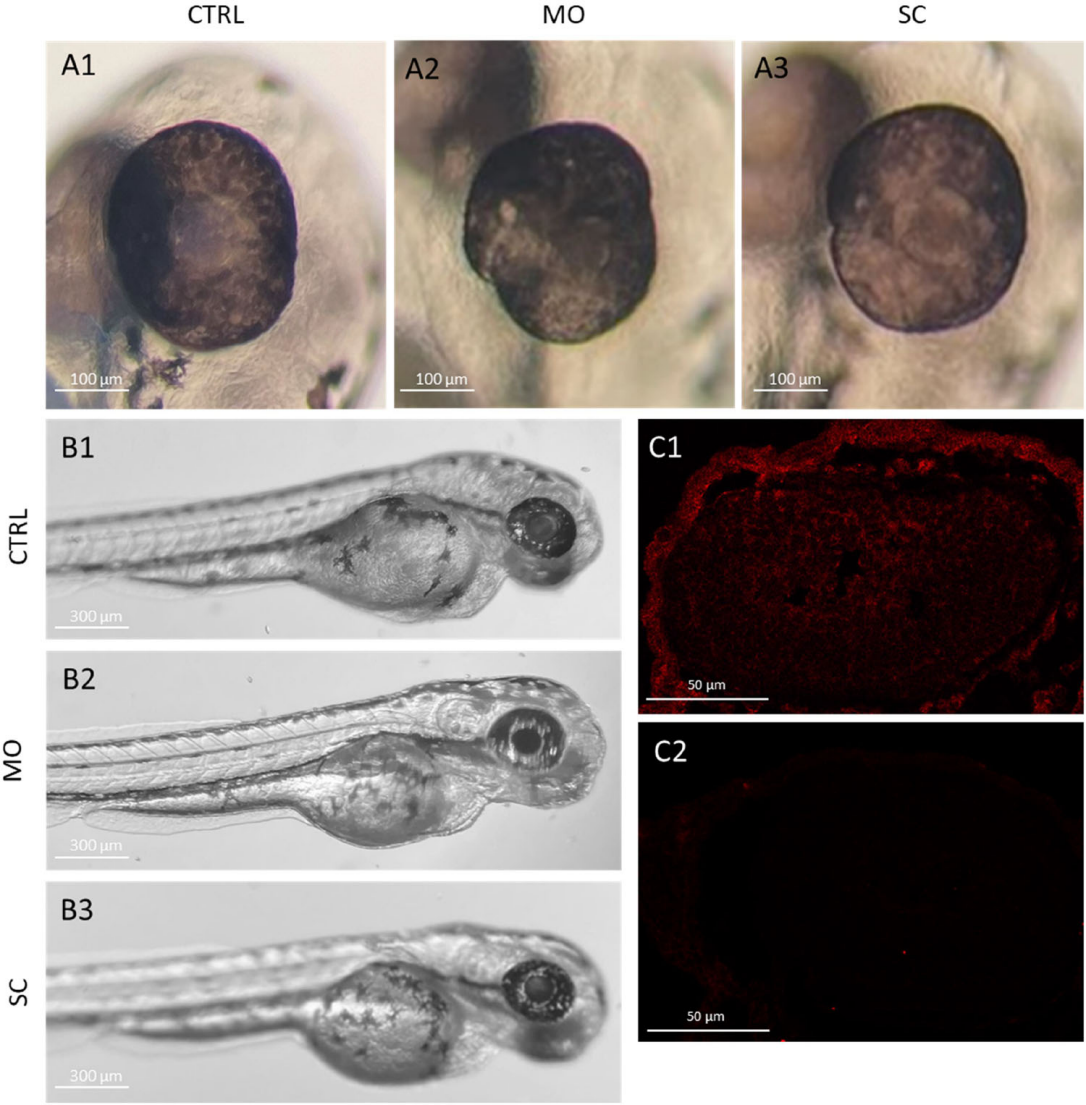
**Morpholino oligo‐mediated knockdown does not influence gross eye size and morphology or overall embryo development but does eliminate Rs1 protein production at 72 hpf**. A: Gross eye morphology and size at 72 hpf were imaged in the sagittal plane of a representative uninjected control (CTRL) (A1), translation‐blocking morpholino oligo (MO)‐injected (A2), and scrambled control oligo (SC)‐injected (A3) embryos under a dissection microscope. Some variations in eye shape were observed in MO‐ and SC‐injected larvae (A2, A3), but no microphthalmia. The scale bars represent 100 µM. B: At 72 hpf, there were no readily discernible differences in the developmental stage between uninjected (B1) MO‐injected (B2) and SC‐injected (B3) siblings. Deformations associated with oligo overdose were very rare at the chosen oligo concentrations (50 ng/µL; less than 1% in 100 injections). C. Rs1 protein presence was observed at 72 hpf in SC larvae (C1) but not in MO‐injected larvae (C2) using IHC. The scale bars represent 50 µm. WT = wild‐type, MO = morpholino‐oligo injected, SC = control injected, hpf = hours post‐fertilization.

The MO concentrations were determined so that no protein was detectable at 72 hpf on IHC, without resulting in larval deformations that are associated with excessive MO concentration, such as enlarged pericardia, microphthalmia, and severe developmental delays (Figure S1C). No microphthalmia was observed in the MO‐ and SC‐injected larvae at the chosen dose concentration (50 ng/µL), but occasional irregularities in eye shape were observed (Figure 5A). This was explored further on IHC. At the chosen concentration, morphants presented normal gross morphology, and deformation occurred in less than 1% of MO‐ or SC‐treated larvae. Representative images of injected and uninjected siblings are shown in Figure 5B. At 72 hpf, no Rs1 protein was detected on IHC in the MO‐treated larvae, unlike their SC‐treated siblings, indicating successful Rs1 knockdown (Figure 5C). Furthermore, Rs1 protein presence was still lagging at 120 hpf compared to SC.

### 3.5 Effect of Rs1a and Rs1b Knockdown on Transcriptome

**FIGURE 6:**
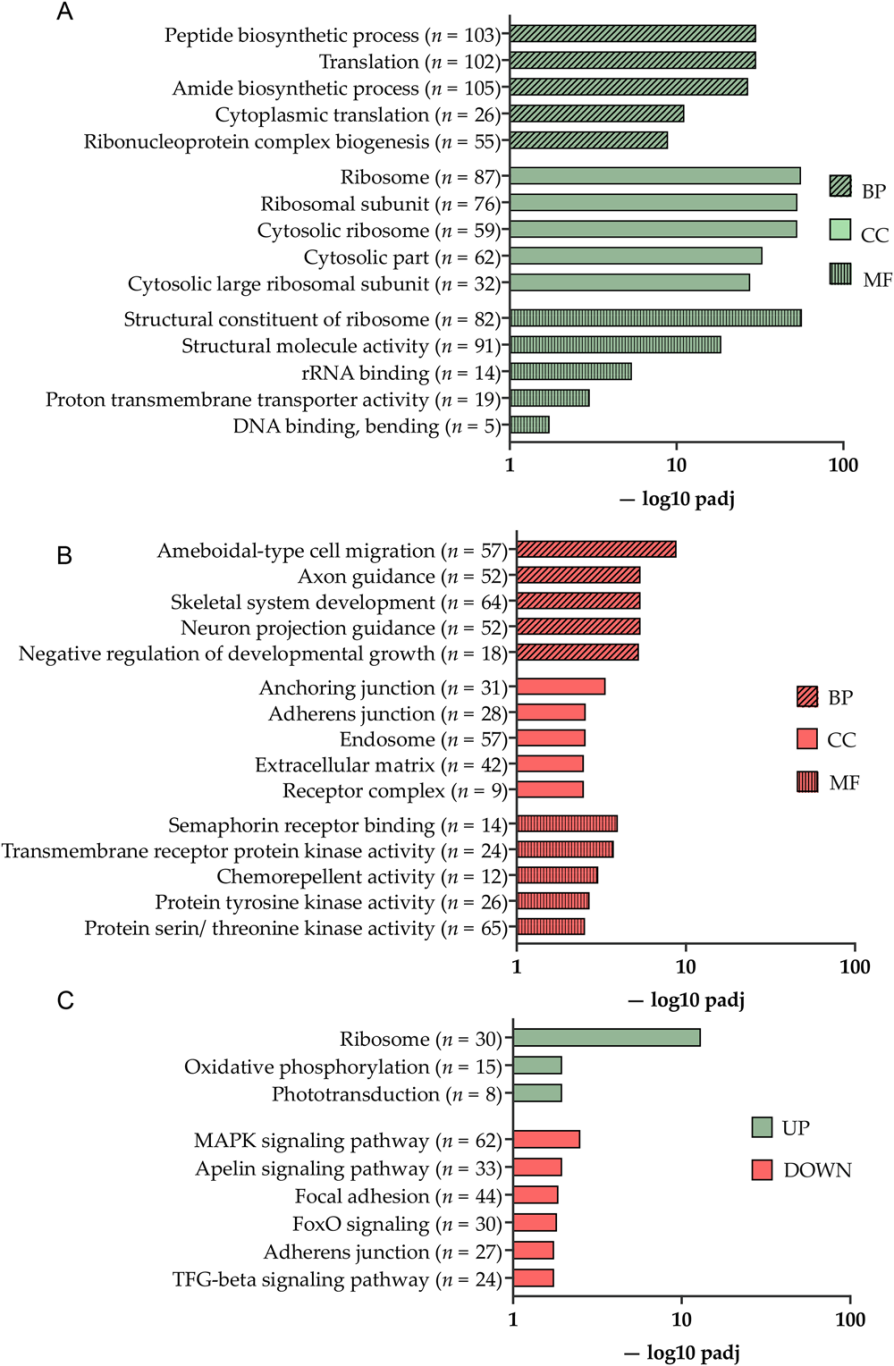
**Delivery of translation‐blocking morpholino oligos targeting *rs1a* and *rs1b* mRNA results in significant downregulation of axon guidance and adherens/anchoring junctions at 72 h post‐fertilization**. Following RNA sequencing, Kyoto Encyclopedia of Genes and Genomes (KEGG) pathway, and Gene ontology (GO) enrichment analyses were performed. A: the top 5 most significantly upregulated GO terms for biological processes (BP; blue bars), cellular components (CC; green bars), and molecular function (MF; red bars). *n* refers to the number of DEGs mapped to the term. B: the top 5 most significantly downregulated GO terms for BP (blue), CC (green), and MF (red). *n* refers to the number of DEGs mapped to the term. C: all significantly upregulated (green bars) and downregulated (red bars) KEGG pathways. *n* refers to the number of DEGs mapped to the pathway. UP = upregulated, DOWN = downregulated. Padj = adjusted *p‐*value.

Following RNA‐Seq, GO analyses per time point were done on three aspects: Biological processes (BP), cellular components (CC), and molecular function (MF). At 48 hpf, only 21 genes were significantly differentially expressed after *p‐*value adjustment. Due to the low number of DEGs, this time point was excluded from further enrichment analysis. At 72 hpf, the most pronounced differences were observed between MO and SC groups, with 727 DEGs post *p*‐value adjustment (186 upregulated and 541 downregulated). At this time point, 71 BP terms, 19 CC terms, and 5 MF terms were statistically significantly enriched in DEGs. Enriched upregulated terms included translation (BP) and ribosome (CC and MF) (Figure 6A). Among the most enriched downregulated BP terms were axon guidance and axon projection, with 52 downregulated DEGs (adj. *p*‐value < 0.005) (Figure 6B). The most notable downregulated genes in this GO term were *vax2* (0.13‐fold downregulated), *disp1* (FC = 0.38), *arnt2* (FC = 0.4), and *ptch2* (FC = 0.4). For CC and BP, respectively, anchoring and adherens junction, and semaphorin receptor binding were noteworthy pathways to be affected. Notable KEGG pathways enriched in downregulated DEGs were focal adhesion and adherens junction (Figure 6C).

**FIGURE 7:**
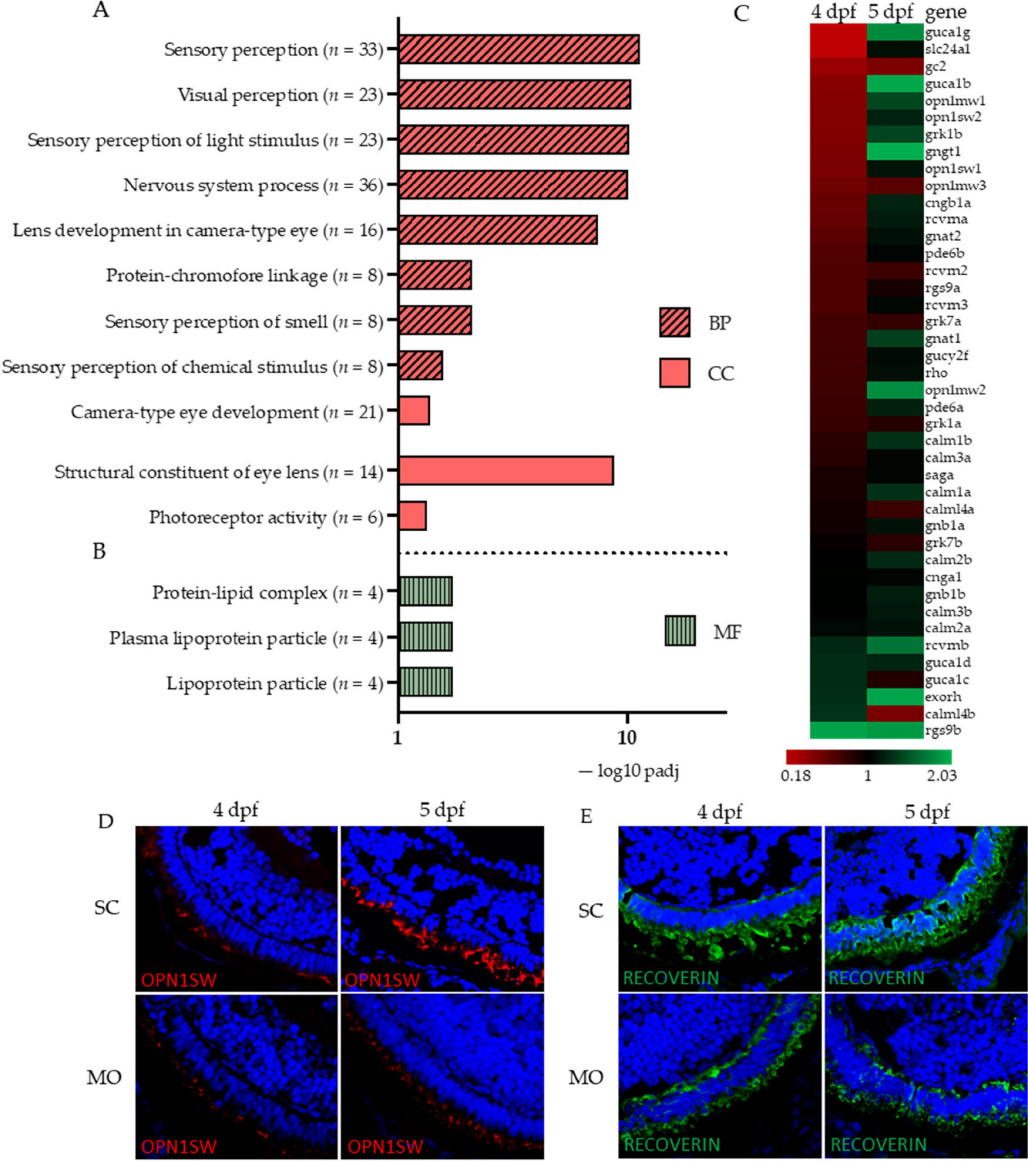
**Delivery of translation‐blocking morpholino oligos (MO) targeting *rs1a* and *rs1b* mRNA results in significant downregulation of visual perception and camera‐type eye development at 96 h post‐fertilization (hpf), as well as impaired photoreceptor development**. Following RNA sequencing, gene ontology (GO) enrichment analyses were performed. A: All significantly downregulated GO terms for biological processes (BP; blue bars) and molecular function (MF; red bars). No terms for cellular components were significantly enriched in differentially expressed genes (DEGs). *n* refers to the number of DEGs mapped to the term. B: All upregulated GO terms for cellular components (CC; green bars). MF and BP did not yield terms significantly enriched in DEGs. *n* refers to the number of DEGs mapped to the term. C: Heatmap of phototransduction‐related genes (derived from Kyoto Encyclopedia of Genes and Genomes [KEGG] pathway). Red and green colors indicate respectively down‐ and upregulation of the genes in MO compared to scrambled (SC). The scale ranges from strongly downregulated (fold change 0.18) to slightly upregulated (fold change 2.0). D: Immunofluorescent staining of blue cone pigment small‐wavelength sensitive opsin (OPN1SW) in 4 and 5 day‐old SC‐ and MO‐injected zebrafish. Padj = adjusted *p*‐value. Dpf = days post‐fertilization.

At 96 hpf, no KEGG pathways were significantly enriched in DEGs. However, GO terms for visual perception, camera‐type eye development, and PR activity are significantly enriched in downregulated DEGs (Figure 7A). Within these terms, key downregulated genes were opsins (*opn4xa*, *opn1sw2*, *opn1sw1*, and *tmtopsa)*, as well as fourteen different crystallin genes. Protein‐lipid complex was identified as a significantly upregulated CC term, due to four lipoprotein genes mapped to this term. These genes were *apoea*, *apobb.1*, *apoa1b*, and *apoa1a*. A heat map of genes mapped to phototransduction is presented in Figure 7C. At 96 hpf, the majority of these genes were downregulated in MO compared to SC, whereas they are not regulated or even upregulated at the 120 hpf timepoint. On IHC, presence of PR proteins small‐wavelength opsin (Opn1sw; Figure 7D) and Recoverin was lower in the MO retina, compared to SC (Figure 7E), and OS were shorter and less developed. This is in line with our transcriptomic findings, which show a downregulation of these genes, with fold changes for opn1sw1, opn1sw2, and recoverin of respectively 0.49, 0.45, and 0.58.

## 4 Discussion

This paper describes the development of the first Rs1‐deficient zebrafish model. First, the wild‐type expression profile of Rs1 was characterized. In wild‐type zebrafish, gene expression of *rs1a* and *rs1b* was first detected using RT‐PCR at 48 hpf, and was maintained up to 120 hpf. *Rs1b* mRNA was found in considerably lower levels than *rs1a*, suggesting that most of the protein derived from the *rs1a* gene may play the more prominent role in zebrafish. At 36 h hpf, retinal progenitor cells give rise to RGC and PR cells, and the GCL starts to take shape (Hu and Easter 1999; Poggi et al. 2005). Rs1 protein presence was first observed on IHC at 48 hpf in the RCL and appeared to increase in quantity up to 120 hpf. Because Rs1 is a secreted protein (Reid et al. 1999), the localization of its synthesis cannot be extrapolated from the location of the protein. PRs exit the cell‐division cycle at 60 hpf and become morphologically distinct at 96 hpf (Avanesov and Malicki 2010). In this time window, Rs1 protein presence appeared to increase significantly. This would place the protein at the right place and time to play a role in retinal development.

Second, we investigated the effect of MO‐mediated r*s1a* and *rs1b* knockdown on retinal development. A temporary blockage of Rs1a and Rs1b protein was achieved through MO‐mediated knockdown. MOs are capable of completely blocking protein translation up to 72 hpf, by which point retinal differentiation is largely completed (Raymond et al. 2006). By injecting the MO in the one‐to‐four cell stage, or up to approximately one hpf, ubiquitous delivery can be achieved (Nasevicius and Ekker 2000). At the chosen dose, we observed little to no protein on IHC for up to four days. By 120 hpf, the presence of Rs1a and Rs1b protein in MO‐injected larvae could once more be detected, but at lower levels than uninjected siblings. Thus, the MOs were able to successfully suppress protein formation past 72 h with no deformations or apparent adverse side effects not related to Rs1 deficiency.

The temporary absence of Rs1 protein resulted in transcriptomic changes in the retina. Following transcriptional analysis, several notable GO terms enriched in downregulated DEGs were identified, including axon guidance at 72 hpf and visual perception at 96 hpf. Visual perception and PR activity being affected at 96 hpf lends credibility to this model being suitable in reflecting Rs1 deficiency. Although Rs1 itself is not a member of the phototransduction cascade, the phototransduction pathway has previously been implicated in a mouse model for RS1 deficiency, as well as an organoid model (Ziccardi et al. 2014; Han et al. 2024). The downregulation of PR genes at 96 hpf is echoed in our IHC findings, where Opn1sw and Recoverin immunostaining showed shorter and less developed PRs in MO‐injected retinae at 4 and 5 dpf. Delays in PR development and other PR defects have been reported in retinal organoids derived from XLRS patients (Duan et al. 2024; Huang et al. 2019). While the downregulation of genes mapped to phototransduction had ceased at 5 dpf in our model, the appearance of the PRs on IHC had not caught up with the SC‐injected fish. We interpret this st in PR development as a delayed structural recovery following the re‐initiation of Rs1 protein function, once the transient effect of the MOs has diminished.

As retinal ganglion cells have been shown to be the first producers of *rs1h* protein in rodents, aberrant RGC axon guidance may be a direct effect of the lack of Rs1 (Liu et al. 2019; Takada et al. 2004). RGCs are not a prevalent target for XLRS research. BPCs, PRs, and Müller glia are the most‐studied cell types in the pathogenesis of XLRS. Furthermore, when RGC abnormalities are observed, their misfunctioning is usually attributed to defective downstream cell types (Liu et al. 2019), when in fact the RGCs may be interesting in their own right. There are some indications of RGC involvement in XLRS. For one, a subset of patients (15.8%) may experience optic disc pallor or nerve fiber layer thinning (62.5%) (Hahn et al. 2022; Genead et al. 2009). Moreover, RS1‐deficient retinal organoids progressively lose expression of the optic atrophy‐related gene *OPA1* (Huang et al. 2019). In several mouse models for XLRS, isolated RGCs show aberrant responses to light, with increased spontaneous firing in some and no response in others (Liu et al. 2019). Furthermore, within the visual perception term, affected genes included several types of crystallins. While crystallins are primarily a component of the lens, they are also present in the retina. In response to injury or stress, retinal crystallin levels may be upregulated (Fort et al. 2009; Piri et al. 2013). However, decreased levels of crystallins in response to retinal injury are also associated with decreased RGC survival (Piri et al. 2013). Furthermore, crystallins may play a role in RGC axon regeneration. Their downregulation in our model underscores the potential detrimental effects of Rs1 deficiency on RGCs in zebrafish.

Taken together, this model shows a mild, transient effect on the transcriptome at 72 and 96 hpf, and altered PR development at 96 and 120 hpf. At 48 hpf, no meaningful differences were found between MO‐ and SC‐injected groups. The number of genes affected was considerably lower than the other conditions, which may be explained by the scarcity of Rs1 protein in the wild‐type situation at that timepoint. We were unable to establish a biological significance for these DEGs based on existing knowledge. The list of DEGs at 48 hpf can be found in Table S3.

Although the classic retinoschisis cavity phenotype was not observed in this model, this is consistent with our expectation that such structural disruptions occur later in development and were not the focus of this early‐stage analysis. Several organoid models for XLRS were similarly unable to show overt structural changes, yet they remain a valuable tool for investigating the underlying molecular mechanisms of the disorder (Han et al. 2024; Duan et al. 2024). Such models can still offer insight into key pathophysiological processes preceding cavity formation, such as gene expression alterations and retinal signaling disruptions. An important drawback of MOs in general is that their effect diminishes over time due to dilution, typically rendering them ineffective after 4–5 days. As the oligo must be injected in the single‐cell stage to limit mosaicism, this renders them ineffective after five days post‐fertilization (Nasevicius and Ekker 2000). In *Rs1h*‐deficient mouse models, restoration of Rs1 protein at early ages was shown to prevent and even reverse most of the disease phenotype (Bush et al. 2016). Thus, even if the follow‐up of the MO‐injected zebrafish extends past five days, the inevitable waning of MO activity will restore Rs1 protein presence and may reverse any changes, especially considering the remarkable regenerative properties of the zebrafish retina. Thus, future studies may benefit from combining or replacing this transient knockdown strategy with stable genetic models, such as CRISPR/Cas9 or ENU‐induced mutants. These models would allow validation of early phenotypes and enable longer‐term analysis of Rs1 deficiency in the zebrafish retina, including investigating the involvement of other cell types at later stages, such as Müller glia.

Nonetheless, this knockdown model has demonstrated that Rs1 deficiency induces alterations in the developing zebrafish retina that can be related to observations in mouse and organoids models for XLRS. Therefore, our current results support the further development of a stable genetic knock‐out model for XLRS in zebrafish. Such a model would enable a more comprehensive exploration of disease progression beyond the five‐day mark, as well as the possibility to evaluate potential therapeutic interventions at earlier stages than rodent models would allow.

## Author Contributions

Conceptualization: I.V., C.K., M.K. and C.B. Methodology: I.V. and J.B. Formal analysis: I.V. Data curation: I.V. Writing—original draft preparation: I.V. Writing—review and editing: I.V., C.K., J.B., M.K. and C.B. Visualization: I.V. Supervision: C.K., M.K. and C.B. Funding acquisition: C.B. All authors have read and agreed to the published version of the manuscript.

## Ethics Statement

All experiments were performed using zebrafish (*Danio rerio*) larvae up to five days post‐fertilisation (dpf), prior to the onset of independent feeding. According to European directive 2019/63/EU and Dutch legislation (Wet op de Dierproeven), zebrafish larvae are not considered protected animals until they reach the stage of independent feeding. Therefore, no ethical approval was required for the experiments described in this study.

## Funding

The funding bodies were not involved in the design and creation of this research. None of the authors report a conflict of interest.

## Conflicts of Interest

The authors declare no conflicts of interest.

## Supporting information




**Supplementary Figure**: dneu23012‐sup‐0001‐FigureS1.docx

**Supplementary Material**: dneu23012‐sup‐0002‐SuppMatt.docx

## Data Availability

The datasets generated and analysed during this study are available from the corresponding author on reasonable request.
