# Supplementary material for: Investigating the Role of Zebrafish Retinoschisin Homologs Rs1a and Rs1b During Retinal Development
Source: Dev Neurobiol. 2025 Oct 31;86(1):e23012. doi: 10.1002/dneu.23012 (PMC12578269; doi:10.1002/dneu.23012)

**Supplementary figure 1: Morphant injected with an excessive MO concentration.** The morphant pictured presented with pericardial edema and defects in development and organ symmetry. At the dose of 100 ng/μL, less than 1 in 100 injected embryos developed this phenotype.


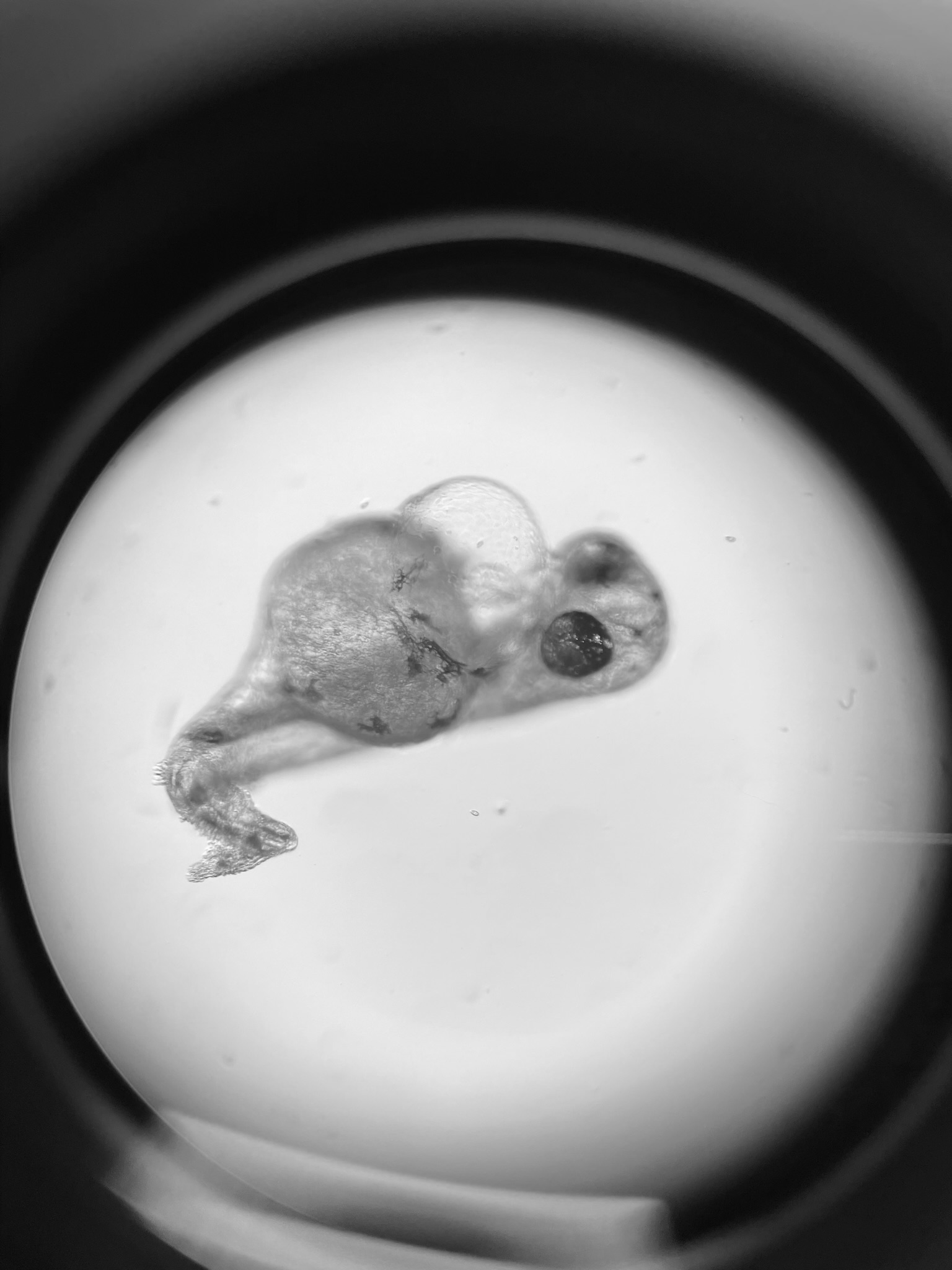

Supplement: Supplementary file 1 — Supplementary Figure: dneu23012‐sup‐0001‐FigureS1.docx [file DNEU-86-0-s002.docx]
