# Supplementary material for: Investigating the Role of Zebrafish Retinoschisin Homologs Rs1a and Rs1b During Retinal Development
Source: Dev Neurobiol. 2025 Oct 31;86(1):e23012. doi: 10.1002/dneu.23012 (PMC12578269; doi:10.1002/dneu.23012)
Supplement: Supplementary file 2 — Supplementary Material: dneu23012‐sup‐0002‐SuppMatt.docx [file DNEU-86-0-s001.docx]

**Supplementary file 1: morpholino oligo sequences and binding region**

Design parameters:

Optimal target regions should adhere as closely as possible to the following design parameters, as outlined by GeneTools, LLC, Philomath, OR, USA. Target regions should be 25 bases in length, lie within the ''5'cap and the first 25 bases of the coding sequence, consist of approximately 50% guanine and cytosine bases, have no secondary structure, and have less than four contiguous guanines.

**S1A. Danio rerio retinoschisin1-a (rs1a; NM_001003438.2) mRNA**

1 aaaaataaa**c atcatggagt acagactgca gaac**acgtta ctgttagcca tcctgctggt

61 gtctcaaggt tttcttggtc tgcaggcaca aacggatggc gaaagcaacg atacatgggc

121 tggaaaatcc tgcaaatgtg attgcgatga ttcctccagc aagatgcttt ccaagggttc

181 atcaagttgg ccacaggaga tgggctgcat gccagagtgt ccctaccata aacctctggg

241 ttttgaggca ggatctgtgg cttcagatca gatcagctgc tctaatgaag accagtacac

301 aggctggttc tcctcctgga cgccaaacag ggcaagacta aatagccaag gatttggatg

361 tgcctggctg tccaagttcc aggacaccag ccagtggctt cagattgacc tgaaggaggt

421 gaaagtggtt tctggtatcc tgacccaggg ccgctgtgac tctgatgagt gggttaccaa

481 gtacactatg cagtaccgca ttaatgacaa tctcaactgg atctactaca aagaccaaac

541 tggaaacaac agggtgttct atgggaactc tgaccgctct tccacagtcc agaacctgct

601 gcgtccacca atcgtagcgc gctacatccg tatcctccct cttggctggc acactcgcat

661 cgccatgcgc ttggagctgc tgctttgcat gaataaatgc acctgagcta ggtaataagc

721 aaagccattc ctaactattt cctgtacacc atccattcat ttctatcaca acatgccaaa

781 gtgaatatca ctagccaatc ttgttttatg tatcatctgg tatggatttc aatcgctttt

841 aattttattt atgtgtttaa tttaacgttc tgcgtctaac tggttgatta tttgattatt

901 cacatatttt tcttcagttc atggaaatta ttgttagatt atgtcatgag taaattactt

961 catgcttttt ttttcatgtc attttttttt taaaagtaaa ataagtattg ctttggcaaa

1021 tcaggtttgt ttacatccaa tgcatgttga agccataatg ttaaaccaga tattcaatcc

1081 gggtatagaa atagggagat ccattggagt tgaactcagt gttgtgttac tgtttttctg

1141 ttgtggtttg tgactgtttt tgcttgtaat tcccactgac cgattccttt ttatattcat

1201 tatgagcctg tactgttaaa ctgtgtttgt ggcttacctg agagacaaca caattgaata

1261 aaggaagtct ttctccgtaa aaaaaaaaaa aaaaaaaaaa aaaccaaaaa aaaaaaaaaa

1321 aaaaaaaaaa

| Oligo | Target | Sequence (''5' to ''3') | Dosage ng/μL |
| --- | --- | --- | --- |
| ZF_RS1a_morpholino | Rs1a | GTTCTGCAGTCTGTACTCCATGATG | 50 |
| ZF_RS1a_control | Rs1a MO control | GTAGTACCTCATGTCTGACGTCTTG | 50 |

**S1B. Danio rerio retinoschisin1-b (rs1a; NM_001004655.1) mRNA**

1 ccagactcca gatctg**ctga** **agatggacag gaggatgctc a**gcgcgctcc tgctgctctc

61 agcgcacgtt ttcattgatg ttcacactca aggggagctg gagataccgt ggccgtatga

121 ggaagagtca gatgtgaagg tagtggagtc agagaaccag accccgagcg gctgcagctg

181 cgcctgtcag aactctccac ccaccagacc tccatcctgg acccccagca gcaccaccac

241 caccacaccc gctacataca gccccatact ggactgcatg cctgagtgtc cataccacag

301 gccactgggg tttgagtccg gctcggtgtt aggagatcag ctcacctgct caaaccagga

361 ccagtacagc ggctggttct cctcctggac ccctaataaa gcccgcctca acagccaagg

421 ctttgggtgt gcgtggctgt cgaaggctca ggacagcagt cagtggctgc aggtggatct

481 gctgcaggtg cgtgtggtgt cggggatcct gtcgcagggc cgctgtgatg cagacgagtg

541 gatcaccaaa tacagcctgc agtaccgctc agaggagcac ctcaactggg tctactacaa

601 agaccagacc ggcaacaacc gggtgtttta cgggaacaca gaccgctcct ctacagtgca

661 gaatctgctg cgtccgccga tcgtggctcg gtacctgcgg atcctcccgc tgggcttcca

721 cacacgcatc gccctgcgca tggagctgct catctgcatg aacaaatgtg gctgatccac

781 acacacacac acacccacac acagagctca gatgagctgt cgagattttc tgttcaatag

841 tttctgtttt ctgttcctga aataaactcc agattactgt aaaaaaaaaa aaaaaaa

| Oligo | Target | Sequence (''5' to ''3') | Dosage (ng/μL) |
| --- | --- | --- | --- |
| ZF_RS1b_morpholino | Rs1b | TGAGCATCCTCCTGTCCATCTTCAG | 50 |
| ZF_RS1b_control | Rs1b MO control | GACTTCTACCTGTCCTCCTACGAGT | 50 |

**S1C: Morphant injected with an excessive MO concentration.** The morphant pictured presented with pericardial edema and defects in development and organ symmetry. At the dose of 100 ng/μL, less than 1 in 100 injected embryos developed this phenotype.


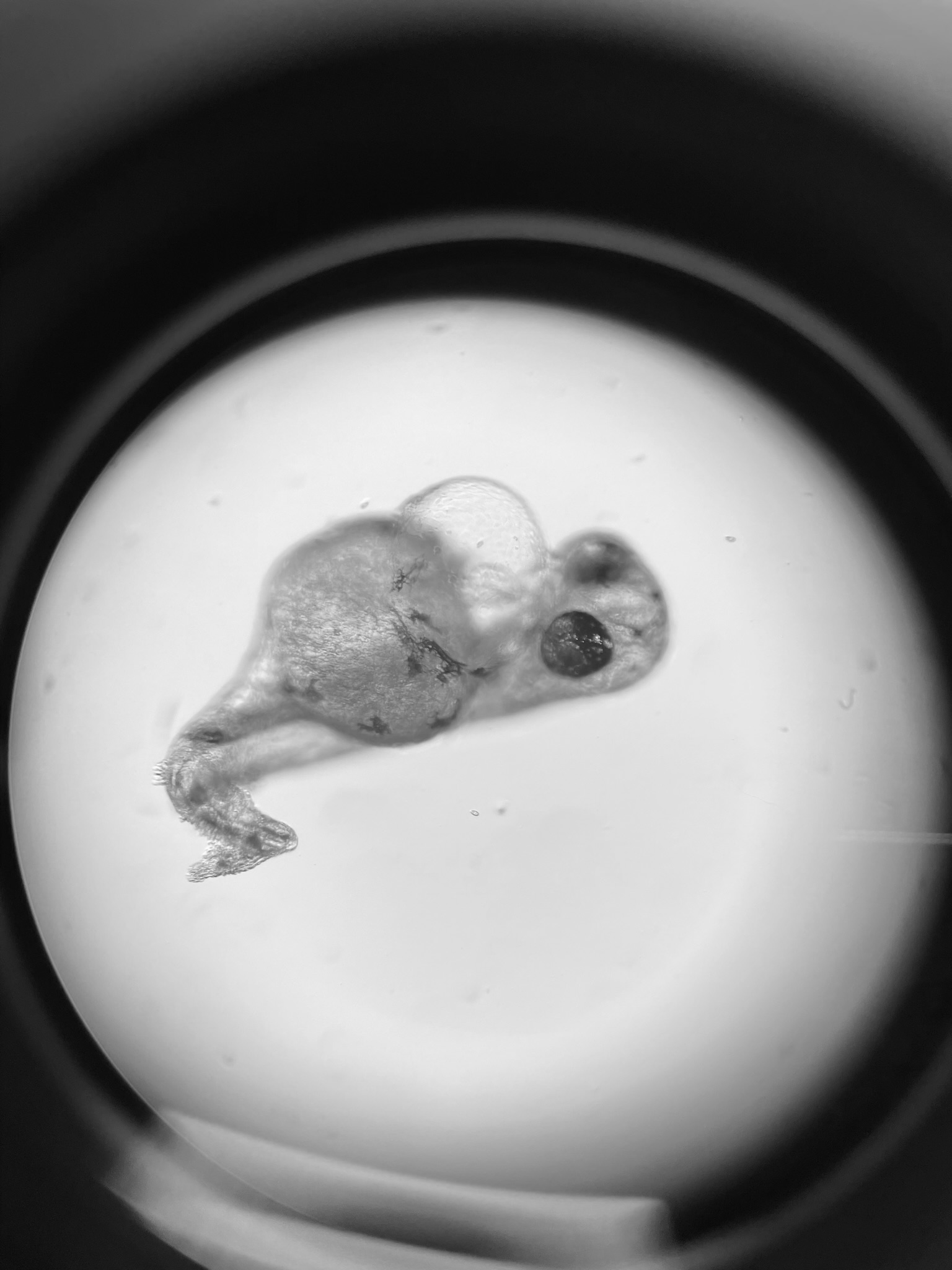


**Supplementary table 1. Primer sequences used for polymerase chain reaction (PCR).** *rs1a* = retinoschisin-1a, *rs1b* = retinoschisin-1b, MO = morpholino, *elfa* = elongating factor 1 alpha, *bactin-1* = beta-actin1, *gfap* = glial fibrillary protein.

| Target | Primer | Sequence (''5' TO ''3') | Application |
| --- | --- | --- | --- |
| rs1a | Forward | AAGTTGGCCACAGGAGATGG | RT-PCR |
|  | Reverse | GAAGAGCGGTCAGAGTTCCC |  |
| rs1b | Forward | AAACCAGGACCAGTACAGCG | RT-PCR |
|  | Reverse | TTGGTGATCCACTCGTCTGC |  |
| MO_rs1a | Forward | CATCATGGAGTACAGACTGC | RT-PCR, sequencing |
|  | Reverse | CACTCATCAGAGTCACAGCG |  |
| MO_rs1b | Forward | CCAGACTCCAGATCTGCTGA | RT-PCR, sequencing |
|  | Reverse | CCTGGTTTGAGCAGGTGAGC |  |
| elfa | Forward | CTTCTCAGGCTGACTGTGC | RT-PCR |
|  | Reverse | CCGCTAGCATTACCCTCC |  |

**Supplementary table 2.** Antibodies used for immunohistochemistry (IHC).

| Target | Clonality | Species | Dilution | Supplier | Number |
| --- | --- | --- | --- | --- | --- |
| Retinoschisin | polyclonal | mouse | 1:100 | Abcam | ab167579 |
| Recoverin | polyclonal | rabbit | 1:200 | Sigma Aldrich | ab5585 |
| Cone arrestin | polyclonal | rabbit | 1:200 | Sigma Aldrich | ab15282 |
| Bllue opsin | monoclonal | mouse | 1:100 | Sigma Aldrich | ab5407 |

**Supplementary table 3.** List of differentially expressed genes (DEGs) at 48 hours post-fertilization (hpf) in zebrafish injected with translation-blocking morpholino oligos versus scrambled controles. Padj = adjusted *p*-value.

| Gene_id | log2FoldChange | pvalue | padj | gene_name |
| --- | --- | --- | --- | --- |
| 336503_1 | 5,355450464 | 4,9E-12 | 1,1E-07 | ifi30 |
| 140533 | -1,752394182 | 1,2E-06 | 0,01339 | ND3 |
| 140535 | -1,88126672 | 2,6E-06 | 0,01464 | ND5 |
| 140531 | -1,763141986 | 3,1E-06 | 0,01464 | ND1 |
| 407073_1 | 3,445114591 | 3,8E-06 | 0,01464 | emc2 |
| 100093702 | 7,847957432 | 4,6E-06 | 0,01464 | si:dkey-121a9.3 |
| 140534 | -1,710643699 | 4,6E-06 | 0,01464 | ND4 |
| 100002814 | 7,639426647 | 7E-06 | 0,01529 | LOC100002814 |
| 140512 | -1,731418118 | 7,1E-06 | 0,01529 | CYTB |
| 140532 | -1,721085026 | 8,1E-06 | 0,01529 | ND2 |
| 140519 | -1,655089391 | 8,2E-06 | 0,01529 | ATP6 |
| 140520 | -1,60722148 | 8,7E-06 | 0,01529 | ATP8 |
| 100334933 | 7,753302899 | 8,9E-06 | 0,01529 | si:ch73-36p18.2 |
| 140538 | -1,850511232 | 9,6E-06 | 0,01529 | ND4L |
| 140541 | -1,734390581 | 1,7E-05 | 0,0246 | COX3 |
| 100334621 | 7,510722397 | 1,8E-05 | 0,0246 | si:ch73-36p18.5 |
| 140523 | -6,990371229 | 2,1E-05 | 0,02729 | trnW |
| 140540 | -1,68457961 | 3,1E-05 | 0,03863 | COX2 |
| 100126106 | 4,898670171 | 3,4E-05 | 0,04031 | ano9a |
| 140513 | -1,634898145 | 3,7E-05 | 0,04105 | trnC |
| 140539 | -1,778038592 | 4,3E-05 | 0,04557 | COX1 |
